# Evaluation of presepsin dynamics in septic patients with and without liver cirrhosis

**DOI:** 10.62838/jccm-2026-0028

**Published:** 2026-07-27

**Authors:** Monica Mlesnite, Petra Fischer, Oana Nicoara-Farcau, Oana Antal, Elena Stefanescu, Horia Stefanescu, Emanuel Palade, Daniela Ionescu, Bogdan Procopet

**Affiliations:** Department of Anesthesia and Intensive Care, Leon Daniello Clinical Hospital of Pneumology, Cluj-Napoca, Romania; Department of Hepatology, Regional Institute of Gastroenterology and Hepatology “Prof. Dr. Octavian Fodor”, Cluj-Napoca, Romania; Department of Hepatology, “Iuliu Hatieganu” University of Medicine and Pharmacy, Cluj-Napoca, Romania; Department of Anesthesia and Intensive Care “Iuliu Hatieganu” University of Medicine and Pharmacy, Cluj-Napoca, Romania; Department of Anesthesia and Intensive Care, Emergency Clinical County Hospital, Cluj-Napoca, Romania; Department of Surgery, “Iuliu Hatieganu” University of Medicine and Pharmacy, Cluj-Napoca, Romania; Department of Surgery, Leon Daniello Clinical Hospital of Pneumology, Cluj-Napoca, Romania; Department of Anesthesia and Intensive Care, Regional Institute of Gastroenterology and Hepatology “Prof. Dr. Octavian Fodor”, Cluj-Napoca, Romania

**Keywords:** sepsis, ICU, cirrhosis, presepsin, Charlson

## Abstract

**Background:**

Sepsis is associated with high short-term mortality, particularly in patients with decompensated liver cirrhosis. Conventional inflammatory biomarkers have limited prognostic accuracy in this population. Presepsin has emerged as a promising biomarker; however, its dynamic behavior in early sepsis remains insufficiently characterized.

**Objectives:**

To evaluate the prognostic value of presepsin dynamics in sepsis, with and without underlying liver cirrhosis, and to compare it with established inflammatory markers.

**Methods:**

We conducted an observational cohort study including two patient groups: patients with sepsis and de-compensated liver cirrhosis (Group A) and patients with sepsis without cirrhosis (Group B). Presepsin levels were measured at admission and after 48 hours, and delta presepsin was calculated. Prognostic performance was assessed using receiver operating characteristic (ROC) analysis and Cox proportional hazards regression. The primary endpoint was 28-day mortality.

**Results:**

In Group A, presepsin measured at 48 hours demonstrated superior prognostic performance compared with baseline levels (AUROC 0.91 vs. 0.78). Changes in presepsin further enhanced risk stratification, with a negative delta associated with improved survival, whereas an increase of ≥500 pg/mL was independently associated with higher mortality. (HR 5, 95% CI:1.64-15.26). In Group B presepsin showed moderate prognostic performance at baseline and 48 hours, whereas delta presepsin demonstrated limited overall accuracy, except in patients with positive delta values. Established biomarkers, including C-reactive protein and procalcitonin, showed inferior prognostic performance compared with presepsin in both cohorts.

**Conclusions:**

Dynamic assessment of presepsin provides clinically relevant prognostic information in sepsis, particularly in patients with decompensated liver cirrhosis. Early changes in presepsin levels may enhance risk stratification beyond conventional inflammatory biomarkers.

## Introduction

Sepsis is a life-threatening condition caused by a dys-regulated immune response to infection, with high short-term mortality [1], especially in older patients and those with comorbidities [2]. Particularly, in patients with liver cirrhosis, the presence of bacterial infections increases mortality fourfold [3].

Early diagnosis and timely initiation of antibiotic therapy are crucial in order to reduce mortality [4,5]. Diagnosing sepsis is challenging due to limitations in clinical examination and in the use of commonly employed biomarkers, such as C-reactive protein (CRP) and procalcitonin (PCT). Consequently, these shortcomings in patients with sepsis have increasingly highlighted the need for more effective diagnostic approaches [6].

Cluster of differentiation 14 (CD14) is a glycoprotein on the cell membranes of monocytes and macrophages that recognizes conserved molecular patterns of both Gram-negative and Gram-positive bacteria. The soluble counterpart of CD14 is named presepsin [7]. Presepsin, a direct indicator of activated monocytes and macrophages in response to pathogens, shows an earlier increase than CRP and PCT, as shown in experimental sepsis and burn studies, and demonstrates a unique capacity to distinguish the severity of sepsis in critically ill patients [8,9].

Increased intestinal permeability in decompensated cirrhosis leads to increased serum lipopolysaccharide (LPS) levels, a virulent part of the outer membrane of Gram-negative bacteria, which triggers the synthesis and secretion of presepsin [10]. Therefore, liver function significantly influences presepsin levels, which are higher in decompensated cirrhosis than in compensated disease [11,12].

Recent studies have also shown a prognostic role for presepsin in patients with severe sepsis both in patients without cirrhosis [13] and in decompensated liver cirrhosis [14].

However, the evolution of presepsin in the early phase of sepsis, particularly its prognostic significance, remains insufficiently characterized. A clearer understanding of this dynamic would be highly valuable for enhancing risk stratification and prognostication, both in patients with or without underlying liver disease.

The primary objective of this study was to assess the prognostic significance of presepsin dynamics in severely ill patients with sepsis across two distinct clinical cohorts: (1) patients with decompensated liver cirrhosis and (2) patients without liver cirrhosis.

As a secondary objective, we aimed to evaluate the prognostic performance of well-established inflammatory biomarkers, namely C-reactive protein and procalcitonin, across the two study cohorts. In addition, we investigated the influence of comorbidity burden, quantified using the Charlson Comorbidity Index, on the prognosis of patients with sepsis, both with and without underlying liver cirrhosis.

## Materials and methods

The study was conducted with respect to the ethical guidelines issued by the 2000 revision (Edinburgh) of the 1975 Declaration of Helsinki and was approved by the Ethical Committee of participating institutions (182/2017, 119/06.02.2015).

### 2.1 Study Design

We conducted an observational cohort study in two tertiary health care centers and utilized a consecutive sampling method to minimize selection bias. The study population consisted of two distinct cohorts. Inclusion and exclusion criteria were defined as follows:

- Group A: Included all consecutive patients diagnosed with sepsis and decompensated liver cirrhosis who were admitted to a tertiary health care center between January 1, 2017, and December 31, 2017.
- Group B: Included all consecutive patients diagnosed with sepsis but without liver cirrhosis who were admitted to the Intensive Care Unit (ICU) in a tertiary health care center between January 1, 2016, and December 31, 2017.

Patients were excluded from both groups if they were younger than 18 years, had incomplete medical records, had a sepsis diagnosis that did not meet the Sepsis-3 criteria, or received treatment that did not comply with current clinical standards.

### 2.2 Definition and application of severity scores

Decompensation of cirrhosis was defined according to existing guidelines as de novo or aggravating ascites, variceal bleeding, or hepatic encephalopathy[15].

For each patient in study group A: MELD-Na score, SOFA score, CLIF-SOFA score (Chronic Liver Failure-Sequential Organ Failure Assessment Score), and a modified Charlson Comorbidity Index were calculated by subtracting 3 points corresponding to the presence of advanced cirrhosis from the original score.

The Charlson Index and the SOFA score were assessed for patients in study group B.

A threshold of 5 points was established to define a severe Charlson Index [16] for both the original and modified versions.

The endpoint was all-cause 28-day mortality.

### 2.3 Type of infections and sepsis definition

#### The types of infections were established according to conventional criteria. [17]

Sepsis was defined according to the Sepsis-3 criteria [1] in patients without liver cirrhosis. In patients with cirrhosis, a modified Sepsis-3 definition was applied in accordance with current guidelines [15], as follows: in patients with a known baseline SOFA score, sepsis was defined as an increase of ≥2 points in the SOFA score in the presence of infection. In patients without a known baseline SOFA score, sepsis was considered when infection was present together with a SOFA score ≥2 and a positive qSOFA score. A positive qSOFA score was defined as the presence of at least two of the following criteria: respiratory rate ≥22/min, systolic blood pressure ≤100 mmHg, or altered mental status (Glasgow Coma Scale <15).

Septic shock was defined as sepsis requiring vasopressors to maintain a mean arterial pressure ≥65 mmHg and a serum lactate level ≥2 mmol/L despite adequate fluid resuscitation

### 2.4 Blood sample collection and biomarker measurement protocol

For each patient in both study groups, 10 mL of venous blood was collected into clot activator tubes and centrifuged at 5000 rpm for 15 minutes, with biomarker analyses conducted within six hours of sample processing.

For study group A, C-reactive protein (CRP) levels were measured using an ELISA kit (Thermo Fisher Scientific, 9811933), and procalcitonin (PCT) levels were determined using a one-step rapid diagnostic test kit (Artron). Baseline presepsin levels were measured at hospital admission from 5 mL of whole blood collected in sodium citrate tubes, using the PATHFAST system in accordance with the manufacturer’s instructions (Mitsubishi Chemical Medience Corporation).

For biomarker assessment in study group B, quantitative PCT measurement was performed using the mini VIDAS B.R.A.H.M.S. system (bioMérieux) based on a fluorescence enzyme immunoassay. Serum CRP levels were determined using an automated immunoassay analyzer (Beckman Coulter, 2015), which employs chemiluminescence detection and magnetic particle separation. Presepsin levels were measured in blood or plasma by automated chemiluminescence immunoassay using the same PATHFAST immunoanalyzer (Mitsubishi Chemical), as applied in study group A.

Presepsin levels were repeated after 48 hours of hospitalisation (48 hours Presepsin). The delta presepsin value (∆ Presepsin) was defined as: 48 hours Presepsin – baseline Presepsin.

### 2.5 Statistical analysis

Statistical analysis was performed using SPSS software version 20 (SPSS Inc., USA) and in R (v4.5.2) using the pROC and survival packages.

Continuous variables were expressed as mean or median, and categorical variables as counts and percentages. Categorical variables were analyzed using Chi-squared test. Continuous variables were analyzed using the unpaired t-test or the Mann-Whitney U test, depending on the population's normality. Receiver operating characteristic (ROC) curves were generated to assess diagnostic performance. Sensitivity (Se), specificity (Sp), and accuracy were calculated. The Youden index was used to identify the optimal threshold on the original dataset. To quantify uncertainty in all performance metrics, internal bootstrap validation was performed with B = 1,000 iterations, reporting 95% confidence intervals for AUROC, sensitivity, and specificity at the fixed Δ500 pg/mL threshold. The random seed was set once prior to all bootstrap analyses to ensure reproducibility independent of group order.

Variables that were significant in the univariable analyses were entered into a multivariable Cox proportional hazards regression model using a backward elimination procedure (entry/removal criteria: p < 0.10 / p > 0.10) to identify variables independently associated with the endpoint. Given the limited number of outcome events, the number of variables that could be included simultaneously in a single model was restricted to minimize the risk of overfitting. Accordingly, we constructed a series of alternative models, each including one presepsin-derived variable alongside the same set of clinical covariates. This strategy enabled the separate estimation of the independent contribution of each presepsin metric while maintaining adherence to the assumptions of multivariable Cox regression. Results were reported as hazard ratios (HRs) with corresponding 95% confidence intervals (CIs). The proportional hazards (PH) assumption was assessed for all Cox regression variables using the Grambsch-Therneau test based on scaled Schoenfeld residuals, implemented via the cox.zph() function in R (survival package). Tests were applied to each covariate individually and as a global test per model. The functional form of continuous presepsin variables (baseline, 48-hour, and delta presepsin) was evaluated using Martingale residuals from reduced Cox models, with LOWESS smoothing applied to assess linearity of the association with the log-hazard.

Kaplan-Meier curves for survival were generated using Log-rank test.

## Results

### 1. Group A

#### 1.1 General characteristics

Forty-one patients with decompensated cirrhosis and sepsis were enrolled in study group A. The demographic data and general characteristics of patients are shown in Table 1.

**Table 1:** Demographic data of study population

**Mean±SD or n (%)**	**Study group A, n=41**	**Study group B, n=81**
**Age**	58±9	63±14.6
**Gender (M)**	33 (80%)	56 (69%)
**Comorbidities**		
Solid neoplasia^*^/lymphoma/leukemia	6/0/0 (14.6%)	12/0/0 (14.8%)
Chronic Pulmonary Disease (CPD)	2 (5%)	11 (13.6%)
Diabetes Mellitus (DM)	9 (22%)	22 (27.2%)
History of stroke/Hemiplegia	3(7.3%)	14/5 (17%/6%)
Chronic Heart Failure (CHF)	6 (10%)	14 (17.3%)
History of myocardial infarct	1(2.4%)	6 (7.4%)
Peripheral vascular disease	0	4 (5%)
Connective tissue disease	0	1 (1.2%)
Peptic ulcer disease	2(4.9%)	8 (10%)
Dementia	0	0
Chronic Renal Disease/severe acute Renal	3 (7.3%)	19 (23.5%)
Disease (creatinine ≥ 3mg/dl)		1 (1.2%)
Chronic Liver Disease (CLD)	41 (100%)	0
Acquired Immunodeficiency Syndrome (AIDS)	0	0
Charlson index^*^ ^*^	3±3	4±2
**Septic shock**	11	52
**MELDNa/Child-Pugh score**	28±7/11±2	-
SOFA score^***^	10±3	9±4
**Type of infection**		
Abdominal	16 (39%)	42 (51%)
Pulmonary	3 (7%)	31 (38%)
Urinary	11 (27%)	0
Soft tissue	0	5 (6%)
Other	11 (27%)	3 (2%)
**Isolated etiological agent**	45	85
	Gram positive-14 (31%)	Gram positive-39 (46%)
	Gram negative-16 (35%)	Gram negative-43 (50%)
	Other-15 (34%)	Other-3 (4%)
**Mortality at 28 days**	24 (58%)	32 (39.5%)

* -Hepatocellular carcinoma in patients with liver cirrhosis

** -modified Charlson index for study group A

*** -CLIF-SOFA score for study group A

Among them, 11 (26.8%) had septic shock. The types of infections were as follows: SBP (n=16, 39%), urinary (n=11, 26.8%), spontaneous bacteremia (n=6, 14.6%), pulmonary (n=3, 7.3%), other (n=5, 12.2%). (Table 1) There were 45 isolated microbiological agents: 14 (31%) Gram-positive, 16 (35%) Gram-negative, 15 (34%) other etiologies. (Table 1)

The mean presepsin value was similar for those with sepsis and septic shock: 2680.8 ± 3589.6 pg/ml and 2536.5 ± 1724.2 pg/ml, respectively. In cases with a Gram-positive etiology, the mean presepsin level was comparable to that observed in Gram-negative infections (1518.9 ± 1276.1 pg/ml vs. 1455 ± 771.7 pg/ml, p = 0.8).

During the follow-up, 24 patients (58.5%) died at 28 days, due to sepsis and multi-organ failure (17, 70.8%), and liver failure (7, 29.2%), respectively.

In patients from the first cohort, the modified Charlson index was not correlated with the endpoint (Pearson’s chi-square, p=0.6).

In patients under 50 years of age, a statistically significant difference in survival was observed between those with cirrhosis and sepsis and those with sepsis but without cirrhosis (Log-rank test, p=0.05), indicating that decompensated cirrhosis, particularly in the context of sepsis, is a strong predictor of poor prognosis. However, among patients aged 50 years or older, this survival difference between the two groups was no longer evident (Log-rank test, p = 0.16).

#### 1.2 Prognostic value of baseline and dynamic presepsin

In study group A, mean baseline presepsin levels were higher in non-survivors than in survivors (3596 ± 4445 vs. 1358 ± 922 pg/mL), although the difference did not reach statistical significance (p =0.07). Baseline presepsin levels demonstrated prognostic value in patients with decompensated cirrhosis, with an area under the receiver operating characteristic curve (AUROC) of 0.78 (95% CI: 0.63–0.92, p = 0.002). A cut-off value of 1303 pg/mL was identified, yielding a sensitivity of 87%, a specificity of 65%, and an overall accuracy of 79%. Presepsin levels measured at 48 hours showed superior prognostic performance, with an AUROC of 0.91 (95% CI: 0.79–1.00, p < 0.0001) and an optimal cut-off value of 1644 pg/mL, corresponding to a sensitivity of 87%, a specificity of 90%, and an accuracy of 87.5%.

After exclusion of patients with severe renal dysfunction (serum creatinine ≥3 mg/dL), the discriminatory performance of presepsin measured at baseline and at 48 hours did not differ significantly, with AUROC values of 0.78 (95% CI: 0.62–0.95, p = 0.005) and 0.88 (95% CI: 0.72–1.00, p = 0.003), respectively.

Delta presepsin values exhibited a markedly skewed distribution and were therefore summarized using the median and interquartile range. The median Δ presepsin at 48 hours was 0 pg/mL (IQR: −265 to 742.5 pg/mL), with values ranging from −1833 to 7888 pg/mL.

Among patients with positive delta presepsin values, prognostic discrimination was moderate (AUROC 0.70, 95% CI: 0.55–0.94; p = 0.5).

The Youden index identified a Δ500 pg/mL threshold as the optimal cut-off within the study cohort; however, this finding should be considered exploratory and hypothesis-generating. This threshold was significantly associated with the outcome (p = 0.02), correctly classifying 71% of fatal events, with a sensitivity of 63% and a specificity of 88%.

**Fig. 1.**
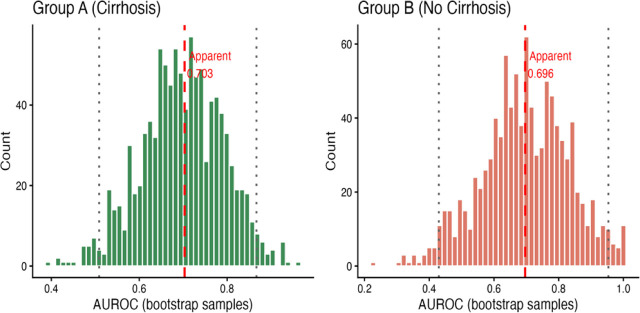
Bootstrap sampling distribution of AUROC (B=1,000 Iteration): group A and B.

**Fig. 2.**
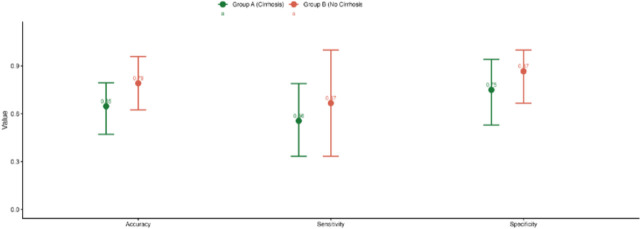
Diagnostic performance of the Δ500 pg/mL threshold for 28-day mortality with 95% bootstrap Confidence Intervals.

To address the potential for optimism bias introduced by deriving the Δ500 pg/mL cut-off via the Youden index in the same dataset, we performed bootstrap internal validation (B = 1,000 iterations), reporting 95% confidence intervals for AUROC, sensitivity, and specificity at the fixed Δ500 pg/mL threshold. In Group A, the AUROC was 0.703 (95% CI: 0.509–0.867), sensitivity 0.556 (95% CI: 0.333–0.790), and specificity 0.750 (95% CI: 0.529–0.941) (Fig. 1A and 2A).

Conversely, a negative delta presepsin value (Δneg) was associated with a favorable outcome, with an observed classification accuracy of 83%. As with the Δ500 threshold, this observation should be interpreted with caution and regarded as hypothesis-generating, pending external validation.

#### 1.3 Prognostic performance of procalcitonin and C-Reactive Protein in decompensated cirrhosis

Procalcitonin demonstrated moderate prognostic performance, with an AUROC of 0.73 (95% CI: 0.57–0.80, p = 0.01). The optimal cut-off value was 0.44 ng/mL, yielding a sensitivity of 82%, a specificity of 58%, and an overall accuracy of 70%.

C-reactive protein showed prognostic performance comparable to procalcitonin, with an AUROC of 0.67 (95% CI: 0.50–0.84, p = 0.05). The optimal cut-off value was 3.53 mg/dL, corresponding to a sensitivity of 87%, a specificity of 53%, and an overall accuracy of 76% (Table 2).

**Table 2.** Prognostic performances of the tested infection markers

	**AUROC (95%CI)**	**Se**	**Sp**	Best cut-off level^*^	**Accuracy**	**P value(AUROC)**
**Study group A**						
CRP (mg/dl)	0.67 (0.50–0.84)	0.87	0.53	3.53	76%	0.05
PCT(ng/ml)	0.73 (0.57–0.80)	0.82	0.58	0.44	70%	0.01
Presepsin_baseline (pg/ml)	0.78(0.63–0.92)	0.87	0.65	1303	79%	0.002
Presepsin48h (pg/ml)	0.91 (0.79–1.00)	0.87	0.90	1644	87%	<0.0001

**Study group B**						
CRP(mg/dl)	0.55 (0.42–0.68)					0.41
PCT(ng/ml)	0.52 (0.39–0.65)					0.68
Presepsin baseline (pg/ml)	0.71 (0.60–0.83)	0.62	0.73	1235	69%	0.001
Presepsin48h (pg/ml)	0.72 (0.52–0.84)	0.61	0.77	1050	72%	0.002

#### 1.4 Factors associated with prognosis

In univariate analysis, CRP, baseline presepsin, 48-hour presepsin, presepsine dynamics (∆neg, ∆500), creatinine value, MELD Na, and CLIF-SOFA, score were all associated with 28-day mortality.

In multivariate Cox regression analysis, among the tested infection markers, adjusted for the MELD score, CLIF SOFA score, and creatinine, only presepsin at 48 hours, ∆500, and ∆neg were independently associated with the outcome (Table 3). Moreover, in multivariate analysis, a Δ presepsin value ≥500 pg/mL (∆500) was associated with a significantly increased hazard of death, with a hazard ratio (HR) of 5 (95% CI: 1.64-15.26). Conversely, a negative Δ presepsin value (∆neg) was associated with a reduced hazard of death, corresponding to an HR of 0.11 (95% CI: 0.02-0.40). No significant violation of the proportional hazards assumption was detected when the Grambsch-Therneau test based on scaled Schoenfeld residuals was applied to all Cox regression models, with results reported per covariate and as a global test.

**Table 3:** Multivariate analysis models were constructed for Study Group A, consisting of four models, each including three variables identified as being associated with survival in univariate analysis. This approach was chosen to minimize collinearity between variables and to account for the relatively limited number of patients with events.

**First model**	**P value**	**HR**	**95%CI**
Presepsin baseline^*^	0.42	1.00	1.00–1.00
MELDNa^*^	0.37	1.04	0.94–1.15
CLIF-SOFA score	<0.001	1.35	1.13–1.61

**Second model**			
Presepsin 48 hours	0.005	1	1.00–1.00
MELDNa^*^	0.50	1.04	0.92–1.17
CLIF-SOFA score^*^	0.67	1.08	0.75–1.55

**Third model**			
∆500	0.005	5.01	1.64–15.26
MELDNa^*^	0.87	0.99	0.88–1.10
CLIF-SOFA score^*^	0.18	1.26	0.89–1.78

**Forth model**			
∆neg	0.001	0.11	0.02–0.40
CLIF-SOFA score	0.04	1.45	1.01–2.08
MELDNa^*^	0.25	0.93	0.83–1.04

* was removed in backward elimination procedure

**Fig. 3.**
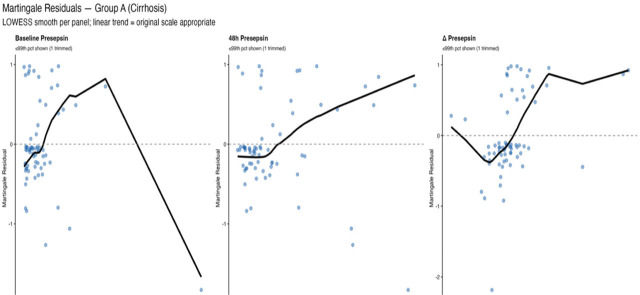
Martingale plots in group A for baseline presepsin, 48h presepsin and Δ presepsin

**Fig. 4.**
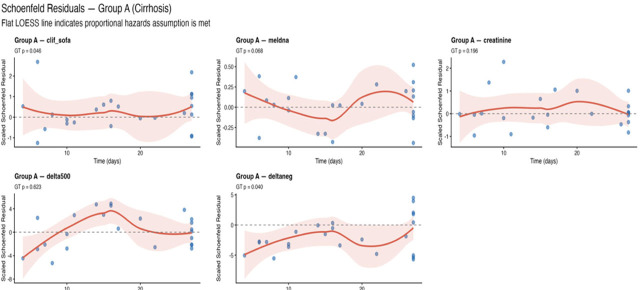
Schoenfeld plots in group A for CLIF-SOFA, MELD-Na, creatinine, Δ presepsin and Δ neg

In Group A, all individual covariate tests and the global test were non-significant (CLIF-SOFA: χ^2^ = 3.19, p = 0.074; MELD-Na: χ^2^ = 2.95, p = 0.086; creatinine: χ^2^ = 1.99, p = 0.158; Δ500: χ^2^ = 0.74, p = 0.390; Δneg: χ^2^ = 3.53, p = 0.060; GLOBAL: χ^2^ = 7.93, df = 5, p = 0.160) (Fig. 3). Schoenfeld residual plots showed no systematic trend over time for any covariate in group B, confirming that the proportional hazards assumption was satisfied throughout the 28-day follow-up period (Fig. 4).

Using the log-rank test, the survival curves of patients with ∆ presepsin ≥ 500 (∆500) are shown in Figure 7.

### 2. Group B

#### 2.1 General characteristics

Eighty-one patients without cirrhosis admitted to the intensive care unit (ICU) were enrolled in study group B.

All patients had sepsis, and more than half of them (64%, n=52) had septic shock. The presence of comorbidities and demographic data of the group are shown in Table 1. The type of infections, in the order of frequency, were as follows: abdominal (n=42, 51%), pulmonary (n=31, 38.3%), soft tissue (n=5, 6.2%), and other etiologies (n=3, 2.43%).

A total of 85 isolated microbiological agents were identified: 39 (45%) were Gram-positive, 43 (50%) were Gram-negative, and 3 (3.5%) were of other etiologies. The mean presepsin value for study group B was 1776±2011.5 pg/ml, which was slightly higher in those with septic shock (2221.6±2286.5 pg/ml). Interestingly, in pure Gram-positive etiologies, the mean presepsin value (1856.5±2001.4 pg/ml) was higher compared to pure Gram-negative etiologies (1497.31±1930.3 pg/ml), although this difference was not statistically significant. (p=0.4)

Thirty-two patients (39.5%) died at 28 days due to: surgical sepsis and multiorgan failure (16, 50%), sepsis and encephalopathy (8, 25%), pulmonary sepsis and multiorgan failure (4, 12,5%) and other causes (4, 12,5%).

A Charlson Comorbidity Index score ≥5, indicative of severe comorbidity burden, was significantly associated with increased short-term mortality in Study Group B (Pearson’s chi-square test, p = 0.01), correctly identifying 69% of patients at risk of death.

#### 2.2 Prognostic value of baseline and dynamic presepsin

A statistically significant difference in baseline presepsin levels was observed between survivors and non-survivors, with higher values recorded in non-survivors (1131.9 ± 1050.8 pg/mL vs. 2763.9 ± 2658.7 pg/mL, respectively; p < 0.0001) (Table 4).

**Table 4:** Mean values of CRP, PCT, presepsin (baseline and at 48 hours) and WBC in survivors vs non-survivors in study group A and B

Variable^*^	**Study group A**			**Study group B**		
	**survivors (n=17)**	**non-survivors (n=24)**	**p**	**survivors (n=49)**	**non-survivors (n=32)**	**p**
Presepsin-baseline (pg/ml)^*^	1358±922	3596±4445	0.07	1131.90±1050.8	2763±2658	<0.0001
Presepsin-48h ^*^ (pg/ml)	1295.63±1281.42	4385.81±4525.38	0.008	930.70±1149.10	2415±2508	<0.0001
∆ Presepsin (median)	−190±337	1088±2378	0.1	−201±962	−324±1927	0.7
CRP (mg/dl) ^*^	4.60±4.02	8.40± 5.94	0.03	27.70±35.30	25.80±11.06	0.6
Procalcitonin ^*^ (ng/ml)	1.77± 3.50	3.49±4.25	0.2	28.40±66.10	27.10±55.20	0.9
WBC (×10^3^/ml) ^*^	11.99±7.55	13.51±7.16	0.5	14.99±9.27	17.45±10.08	0.2

* (mean ± SD)

In patients with sepsis without liver cirrhosis, baseline presepsin demonstrated good prognostic performance, with an area under the receiver operating characteristic curve (AUROC) of 0.72 (95% CI: 0.60–0.83, p = 0.001). The optimal cut-off value was 1235 pg/mL, yielding a sensitivity of 63.3%, a specificity of 73.4%, and an overall accuracy of 69.1%. Presepsin measured at 48 hours showed comparable prognostic performance, with an AUROC of 0.72 (95% CI: 0.58–0.84, p = 0.002). According to the Youden index, a cut-off value of 1050 pg/mL was identified, corresponding to a sensitivity of 61.5%, a specificity of 78.5%, and an accuracy of 72%.

Subgroup analysis restricted to patients with septic shock revealed that baseline presepsin maintained moderate prognostic value, with an AUROC of 0.71 (95% CI: 0.57–0.85, p = 0.007). A cut-off value of 1570 pg/mL was associated with a sensitivity of 63%, a specificity of 68%, and an accuracy of 65%. The prognostic performance of presepsin measured at 48 hours was slightly lower in this subgroup, with an AUROC of 0.64 (95% CI: 0.47–0.81, p = 0.09).

After exclusion of patients with severe renal dysfunction (serum creatinine ≥3 mg/dL), the discriminatory performance of presepsin decreased, with AUROC values of 0.62 for baseline measurements and 0.64 for measurements obtained at 48 hours.

Delta presepsin values exhibited a markedly skewed distribution, with a median of −163.0 pg/ml (IQR: −450.5 to 95.5) and a range from −5259.0 to 4002.0 pg/ml.

Overall, delta presepsin showed poor prognostic performance in patients without cirrhosis (AUROC = 0.49), which remained similarly low in patients with septic shock (AUROC = 0.44) and after exclusion of patients with severe renal dysfunction (AUROC = 0.54).

However, when the analysis was limited to patients with positive delta presepsin values, prognostic performance improved, with an AUROC of 0.69 (95% CI: 0.43–0.95, p = 0.1).

In this context, the Youden index identified an optimal cut-off value of 500 pg/mL (Δ500), which was significantly associated with the outcome (p = 0.01). Using this threshold, 70% of patients with a fatal outcome were correctly identified, with very few false positives (specificity >90%).

The bootstrap internal validation (B = 1,000 iterations), reporting 95% confidence intervals for AUROC, sensitivity, and specificity at the fixed Δ500 pg/mL threshold resulted in an AUROC of 0.696 (95% CI: 0.430–0.954), sensitivity 0.667 (95% CI: 0.333–1.000), and specificity 0.867 (95% CI: 0.667–1.000). (Fig 1B and 2B)

#### 2.3 Prognostic performance of procalcitonin and C-Reactive Protein in patients without cirrhosis

In patients without cirrhosis the prognostic performance of the other biomarkers, CRP and PCT, was lower, with AUROCs of 0.55 (95%CI: 0.42-0.68, p=0.4) and 0.52 (95%CI: 0.39-0.65, p=0.6), respectively (Table 2).

#### 2.4 Factors associated with prognosis

**Fig. 5.**
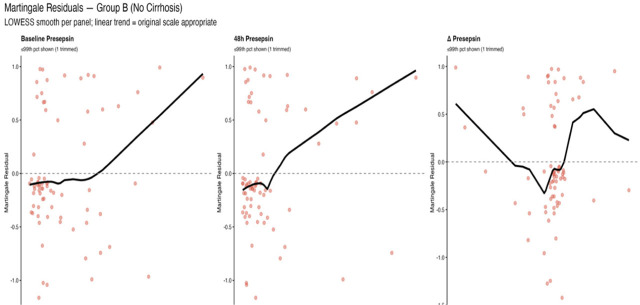
Martingale plots in group B for baseline presepsin, 48h presepsin and Δ presepsin

**Fig. 6.**
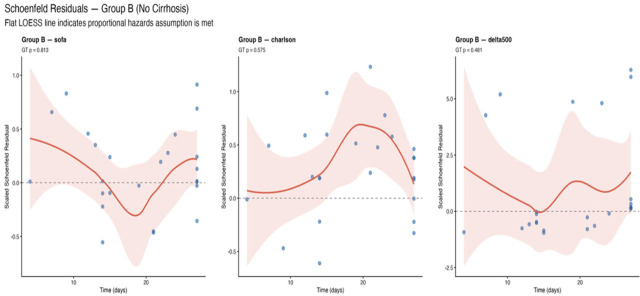
Schoenfeld plots in group B for SOFA score, Charlson and Δ presepsin.

In univariate analysis, baseline presepsin, presepsin at 48 hours, Δ 500, the Charlson comorbidity index, and the SOFA score were all significantly associated with 28-day mortality. In multivariate Cox regression analysis, three models—each comprising three of the aforementioned variables—were evaluated (Table 5). Presepsin at 48 hours and Δ500 emerged as independent predictors of short-term mortality. Among the variables included in the multivariate analysis, Δ500 demonstrated the highest hazard ratio (HR = 2.69, 95%CI: 1.06-6.81). All tests (Grambsch-Therneau test) were similarly non-significant (SOFA: χ^2^ = 0.01, p = 0.916; Charlson: χ^2^ = 0.82, p = 0.366; Δ500: χ^2^ = 0.65, p = 0.420; GLOBAL: χ^2^ = 1.43, df = 3, p = 0.699) (Fig.5) and Schoenfeld residual plots showed no systematic trend over time for any covariate (Fig. 6), confirming that the proportional hazards assumption was satisfied.

**Table 5:** Models for multivariate analysis Study group B

**First model**	**P value**	**HR**	**95%CI**
Presepsin baseline	0.005	1.00	1.00–1.00
Charlson index	<0.0001	1.34	1.15–1.57
SOFA score^*^	0.11	1.10	0.97–1.25

**Second model**			
Presepsin 48 hours	0.002	1.00	1.00–1.00
Charlson index	0.009	1.30	1.10–1.54
SOFA score^*^	0.17	1.09	0.96–1.24

**Third model**			
∆ 500	0.01	2.69	1.06–6.81
Charlson index	0.001	1.33	1.12–1.59
SOFA score	0.03	1.13	1.00–1.30

* was removed in backward elimination procedure

The reduced survival of patients with an increase in presepsin of at least 500 pg/mL at 48 hours, compared to baseline, is illustrated by Kaplan-Meier curves (Log-rank, p = 0.004) (Figure 8).

**Fig. 7.**
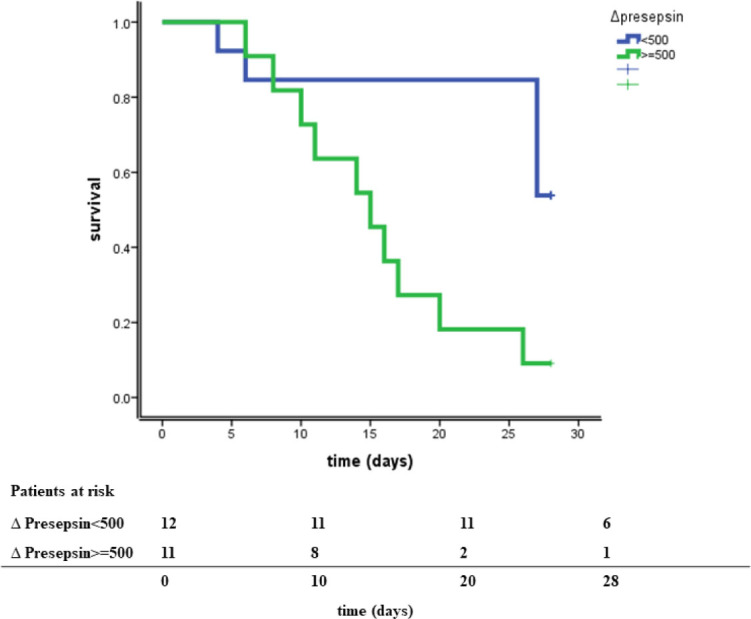
Kaplan Maier curve for study group A. Curves were compared using a Log-rank test

**Fig. 8.**
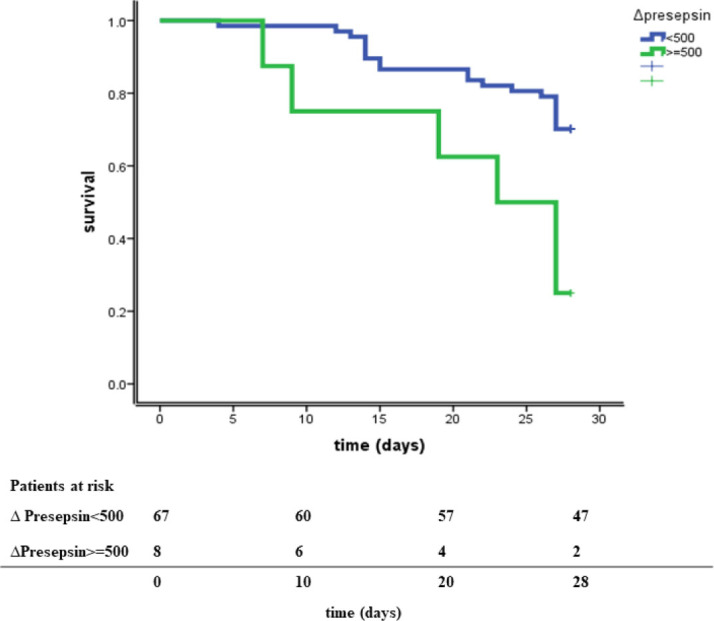
Kaplan Maier curve for study group B. Curves were compared using a Log-rank test

## Discussion

In this observational cohort study, we evaluated the prognostic value of presepsin dynamics in patients with sepsis, with and without underlying decompen-sated liver cirrhosis, and compared its performance with established inflammatory biomarkers. Our results suggest that presepsin, particularly when assessed dynamically at 48 hours and through delta changes, may provide additional prognostic information, especially in patients with decompensated cirrhosis. Although presepsin has attracted growing scientific interest, it is not currently included in the 2021 Surviving Sepsis Campaign guidelines, where procalcitonin remains the gold-standard biomarker for the diagnosis of sepsis [5]. Numerous studies have investigated presepsin as a bio-marker for sepsis and septic shock, indicating potential diagnostic and prognostic value and supporting its role as a complement to established biomarkers.

In patients with cirrhosis (Group A), presepsin measured at 48 hours exhibited superior prognostic performance compared with baseline levels, as reflected by higher AUROC values and improved diagnostic accuracy. This finding supports previous evidence suggesting that dynamic biomarker assessment may be more informative than single-time-point measurements in critically ill patients [18,19]. The enhanced prognostic accuracy at 48 hours likely reflects both disease severity and early response to therapy, capturing the evolving host–pathogen interaction during sepsis. Bootstrap internal validation (B = 1,000) at the fixed Δ500 pg/mL threshold confirmed moderate discriminative ability across the overall cohort and both subgroups, with AUROC point estimates ranging from 0.696 to 0.708. The wide confidence intervals, most pronounced in Group B (AUROC 0.415–0.941; sensitivity 0.333–1.000), reflect the inherent imprecision of performance estimates in small samples, particularly with only 9 events in 24 patients. These findings support the internal consistency of the Δ500 pg/mL threshold but do not substitute for external validation.

Delta presepsin further improved prognostic stratification in cirrhotic patients. A negative delta presepsin value was associated with improved survival, whereas an increase of at least 500 pg/mL (Δ500), derived within the present cohort, was independently associated with a significantly increased risk of death in multivariable analysis. These findings should be considered exploratory and hypothesis-generating, and the proposed Δ500 threshold requires prospective validation in independent datasets.

The prognostic utility of presepsin in cirrhosis should be interpreted in the context of the underlying pathophysiology of chronic liver disease. Previous studies have demonstrated elevated presepsin levels in patients with cirrhosis compared to healthy controls, likely attributable to bacterial translocation, a hallmark of advanced cirrhosis [20]. Efremova et al. found that presepsin levels in blood increase as cirrhosis worsens, as assessed by the Child-Pugh score [20]. This mechanism likely explains the higher baseline presepsin values observed in cirrhotic patients and highlights the added value of dynamic measurements over absolute cut-off values in this population.

The microbiological characteristics of the causative pathogen do not appear to significantly influence presepsin levels, which remain comparable in infections caused by Gram-positive and Gram-negative bacteria, both in patients with and in patients without cirrhosis. In line with these findings, Lee et al. concluded that the primary driver of variability in presepsin levels during bloodstream infections may be the pathogen's immunogenicity rather than the presence of lipopolysaccha-ride (LPS) [21].

In patients without cirrhosis (Group B), presepsin showed some association with the outcome at baseline and at 48 hours, whereas delta presepsin demonstrated more limited overall discriminatory performance. However, when the analysis was restricted to patients with positive delta presepsin values, prognostic accuracy improved, suggesting that higher presepsin levels may help identify a subgroup of patients with a greater inflammatory burden and potentially worse outcomes. These findings are consistent with previous studies reporting variable prognostic performance of presepsin in heterogeneous septic populations [22].

Established inflammatory biomarkers, such as C-reactive protein and procalcitonin, demonstrated variable prognostic performance across the studied populations, with more modest discrimination observed in patients with cirrhosis and less consistent performance in those without cirrhosis. While these findings are broadly in line with previous reports suggesting limitations of conventional biomarkers in severe sepsis, they should be interpreted with caution given methodological differences in biomarker measurement between the two cohorts. In particular, the use of different analytical platforms may have introduced inter-assay variability, potentially affecting absolute values, derived cut-off thresholds, and estimates of prognostic accuracy. Procalcitonin levels were determined using two different analytical methods: the Artron Procalcitonin Rapid Test and the VIDAS B.R.A.H.M.S. assay performed on the mini VIDAS platform. The Artron system provides semi-quantitative results and may have lower analytical sensitivity and precision than automated laboratory assays, which may limit the accurate assessment of low PCT concentrations. Furthermore, PCT concentrations may be elevated in non-infectious conditions such as major surgery, trauma, or systemic inflammatory states, potentially reducing specificity for bacterial infection. Conversely, PCT levels may remain low during the early stages of infection or in localized infections. Regarding CRP, concentrations were determined using two different analytical methods: an ELISA kit (Thermo Fisher Scientific, catalog no. 9811933) and an automated immunoassay analyzer (Beckman Coulter, 2015). ELISA-based measurements are more susceptible to operator-dependent variability and longer processing times, while automated chemiluminescent assays may be influenced by instrument-specific calibration and reagent variability. From a clinical perspective, CRP is a non-specific inflammatory biomarker, and elevated concentrations may occur in a variety of inflammatory conditions, including trauma or other non-infectious processes, which may limit its specificity for infection or sepsis.

Renal dysfunction represents an important confounding factor in presepsin interpretation, as impaired renal clearance is associated with elevated circulating levels. In our study, excluding patients with severe renal dysfunction reduced the discriminatory performance of presepsin, underscoring the need for cautious interpretation of absolute presepsin values in patients with acute kidney injury [23]. This finding further supports the relevance of dynamic changes rather than static thresholds. At the same time, renal dysfunction was incompletely characterized in our study, primarily due to the limited number of included patients. This constraint did not allow for a reliable evaluation of presepsin performance across different stages of renal impairment. Larger cohorts will be required to more accurately characterize the influence of renal function on presepsin levels and its prognostic performance across the full spectrum of renal dysfunction.

In our study, comorbidity burden, as measured by the Charlson Comorbidity Index, was significantly associated with prognosis in patients without cirrhosis. However, this association was not evident in patients with decompensated cirrhosis, suggesting that in this group, the severity of liver disease may have a more decisive impact on outcomes than coexisting comorbidities.

Several limitations of this study should be acknowledged. The small sample size, particularly in subgroup analyses, may have limited statistical power. Moreover, this limitation also applied to exploratory Cox regression models, where only a limited number of variables were tested. In particular, the limited number of patients with septic shock precluded the performance of a dedicated subgroup analysis among patients with liver cirrhosis. The observational design precludes causal inference, and the use of different analytical platforms for biomarker measurement across cohorts may have introduced analytical variability. Patients were recruited from multiple institutions, where differences in treatment protocols may have influenced outcomes. A further limitation of this study was the potential optimism bias related to deriving the Δ500 pg/mL cut-off within the same dataset. Although bootstrap internal validation was performed, the estimated performance metrics remain subject to uncertainty given the sample size. Therefore, the Δ500 pg/mL threshold should be considered exploratory and requires external validation in independent cohorts. Nevertheless, the consistency of findings across multiple analytical approaches strengthens the validity of our results.

## Conclusion

Overall, our data support the clinical relevance of presepsin dynamics as a prognostic marker in sepsis, particularly in patients with decompensated cirrhosis. Dynamic assessment of presepsin over the first 48 hours may enhance early risk stratification and aid clinical decision-making in this vulnerable population.
